# Supplementary material for: Association between health-related hope and adherence to prescribed treatment in CKD patients: multicenter cross-sectional study
Source: BMC Nephrol. 2020 Oct 31;21:453. doi: 10.1186/s12882-020-02120-0 (PMC7603681; doi:10.1186/s12882-020-02120-0)
Supplement: Supplementary file 1 — Additional file 1: Table S1. English version of the 18-item Health-Related Hope scale. [file 12882_2020_2120_MOESM1_ESM.docx]

# **Table S1. English version of the 18-item Health-Related Hope scale.**

| Stem | Please answer the questions below while keeping in mind how you feel about your future health. |
| --- | --- |
| Question 1 | I think I’ll still be able to continue doing enjoyable things in the future. |
| Question 2 | I will probably be able to discover some meaning to my life. |
| Question 3 | I will probably be able to work, to the best of my ability, to find a sense of purpose in life. |
| Question 4 | I will probably be able to live each day to its fullest. |
| Question 5 | I feel that I can continue to experience a sense of fulfillment in my daily life. |
| Question 6 | Even if I should feel down due to my illness, I could probably turn my feelings around. |
| Question 7 | I feel I can adjust my health goals in a way that is consistent with my actual disease condition. |
| Question 8 | I can probably alter my goals depending on changes in my illness or symptoms. |
| Question 9 | Even if my health condition keeps me from achieving my present goals, I will probably be able to find a new goal. |
| Question 10 | I can probably develop a personal lifestyle strategy for dealing with my disease condition. |
| Question 11 | I will probably be able to find a way to keep my illness from worsening. |
| Question 12 | I will probably be able to continue performing my role in society. |
| Question 13 | My disease experience will probably encourage those around me to be mindful of their own health. |
| Question 14 | I feel I can deepen my relationships with my friends. |
| Question 15 | Those around me will probably go along with any changes in my mood. |
| Question 16 | Those around me will probably continue to treat me the same way they always have. |
| Response options for Questions 1 through16 | I don't feel that way at all  I feel that way a little  I feel that way somewhat  I feel that way strongly |

| Question regarding family | Do you have any family? |
| --- | --- |
| Response options | Yes  No |
| Question 17 | I’ll probably be able to continue my usual role in support of my family. |
| Question 18 | I feel that I’ll continue to have a good relationship with my family. |
| Response options for Questions 17 and 18 | I don't feel that way at all  I feel that way a little  I feel that way somewhat  I feel that way strongly |

Before using this instrument, please register through https://www.sf-36.jp/.

In addition, please cite this article as a reference:

Fukuhara S, Kurita N, Wakita T, Green J, Shibagaki Y.

A scale for measuring health-related hope: its development and psychometric testing.

Annals of Clinical Epidemiology 2019;1(3):102-119
