# Supplementary material for: Association between health-related hope and adherence to prescribed treatment in CKD patients: multicenter cross-sectional study
Source: BMC Nephrol. 2020 Oct 31;21:453. doi: 10.1186/s12882-020-02120-0 (PMC7603681; doi:10.1186/s12882-020-02120-0)
Supplement: Supplementary file 2 — Additional file 2: Table S2. Japanese version of the 18-item Health-Related Hope scale. [file 12882_2020_2120_MOESM2_ESM.docx]

# **Table S2. Japanese version of the 18-item Health-Related Hope scale.**

| Stem | これからのご自身の健康状態をふまえて、以下の質問にお答えください。 |
| --- | --- |
| Question 1 | これからも楽しみを持ち続けることができると思う。 |
| Question 2 | 自分が生きる意味を見つけられるだろう。 |
| Question 3 | 自分のできる範囲で、生きがいを見つけられるだろう。 |
| Question 4 | 毎日を大切に過ごしていけるだろう。 |
| Question 5 | 日々の暮らしの中で、生きがいを感じていけると思う。 |
| Question 6 | 病気によって気分が落ち込むことがあっても、気持ちを切り替えられるだろう。 |
| Question 7 | 病状に応じて、現実的な健康上の目標を決められると思う。 |
| Question 8 | 私は、病気や症状の変化に応じて、目標を修正できるだろう。 |
| Question 9 | 病気によって目標が達成できなくなっても、また新たな目標を見つけられるだろう。 |
| Question 10 | 病状に併せて、自分なりに生活の工夫ができるだろう。 |
| Question 11 | 病気を悪化させないための方法を探すことができるだろう。 |
| Question 12 | 現在、社会の中で果たしている役割をこれからも続けられるだろう。 |
| Question 13 | 私の病気の体験を知ることで、周囲の人も健康を気遣うようになるだろう。 |
| Question 14 | 友人とより良い関係を作る事ができると思う。 |
| Question 15 | 私の周囲の人は私の気分転換に付き合ってくれるだろう。 |
| Question 16 | 私の周囲の人はこれからも今まで通り接してくれるだろう。 |
| Response options for Questions 1 through16 | 全くそう思わない  少しそう思う  ある程度そう思う  とてもそう思う |

| Question regarding family | ご家族はいらっしゃいますか？ |
| --- | --- |
| Response options | はい  いいえ |
| Question 17 | 現在、家族の中で果たしている役割をこれからも続けられるだろう。 |
| Question 18 | 今後も家族との良い関わりが続くと思う。 |
| Response options for Questions 17 and 18 | 全くそう思わない  少しそう思う  ある程度そう思う  とてもそう思う |

Before using this instrument, please register through https://www.sf-36.jp/.

In addition, please cite this article as a reference:

Fukuhara S, Kurita N, Wakita T, Green J, Shibagaki Y.

A scale for measuring health-related hope: its development and psychometric testing.

Annals of Clinical Epidemiology 2019;1(3):102-119
