# Supplementary material for: Association between health-related hope and adherence to prescribed treatment in CKD patients: multicenter cross-sectional study
Source: BMC Nephrol. 2020 Oct 31;21:453. doi: 10.1186/s12882-020-02120-0 (PMC7603681; doi:10.1186/s12882-020-02120-0)
Supplement: Supplementary file 3 — Additional file 3: Figure S1. Conceptual framework used in regression analyses. [file 12882_2020_2120_MOESM3_ESM.docx]

# **Figure S1. Conceptual framework used in regression analyses.**

For analysis 1, we examined the association of HR-Hope scores with (1-i) the perceived burden of fluid restriction and (1-ii) the perceived burden of diet restriction. For analysis 2, we examined the association of HR-Hope scores with (2-i) systolic blood pressure and (2-ii) diastolic blood pressure. For analysis 3, we examined the association of HR-Hope scores with (3-i) serum phosphorus levels, and (3-ii) serum potassium levels. For those three analyses, HR-Hope was considered to be an exposure. We also examined whether or not HR-Hope was associated with the stage of kidney disease. For that analysis, HR-Hope was considered to be an outcome.
