# Supplementary material for: Association between health-related hope and adherence to prescribed treatment in CKD patients: multicenter cross-sectional study
Source: BMC Nephrol. 2020 Oct 31;21:453. doi: 10.1186/s12882-020-02120-0 (PMC7603681; doi:10.1186/s12882-020-02120-0)
Supplement: Supplementary file 4 — Additional file 4: Table S3. The associations between HR-Hope and blood pressures. [file 12882_2020_2120_MOESM4_ESM.docx]

# **Table S3. Associations between HR-Hope and blood pressure^a^**

|  | SBP (N=429), Mean difference, mmHg (95%CI) | | | | | | | | | | | | | | |  | DBP (N=408), Mean difference, mmHg (95%CI) | | | | | | | | | | | | | | |
| --- | --- | --- | --- | --- | --- | --- | --- | --- | --- | --- | --- | --- | --- | --- | --- | --- | --- | --- | --- | --- | --- | --- | --- | --- | --- | --- | --- | --- | --- | --- | --- |
|  | Unadjusted | | | | | Adjusted 1^b^ | | | | | Adjusted 2^c^ | | | | |  | Unadjusted | | | | | Adjusted 1^b^ | | | | | Adjusted 2^c^ | | | | |
| **HR-Hope** |  | | | | |  | | | | |  | | | | |  |  | | | | |  | | | | |  | | | | |
| *per 10 point* | **-2.53** | | | | | **-1.72** | | | | | **-1.87** | | | | |  | **-0.92** | | | | | -0.45 | | | | | -0.57 | | | | |
|  | **(** | **-3.76** | **-** | **-1.30** | **)** | **(** | **-2.95** | **-** | **-0.49** | **)** | **(** | **-3.08** | **-** | **-0.65** | **)** |  | **(** | **-1.75** | **-** | **-0.09** | **)** | ( | -1.24 | - | 0.34 | ) | ( | -1.37 | - | 0.23 | ) |
| *per 1 SD* | **-4.72** | | | | | **-3.21** | | | | | **-3.49** | | | | |  | **-1.71** | | | | | -0.83 | | | | | -1.06 | | | | |
|  | **(** | **-7.03** | **-** | **-2.42** | **)** | **(** | **-5.51** | **-** | **-0.92** | **)** | **(** | **-5.75** | **-** | **-1.22** | **)** |  | **(** | **-3.26** | **-** | **-0.17** | **)** | ( | -2.30 | - | 0.64 | ) | ( | -2.54 | - | 0.42 | ) |

^a^General linear models were used to estimate mean differences in blood pressure.

^b^Adjusted for age, gender, stage of renal disease, performance status, presence of family, work status, and number of categories of prescribed antihypertensives

^c^Adjusted for the covariates listed in footnote b, and also for primary renal disease, diabetes, coronary artery disease, and cerebrovascular disease
