# Supplementary material for: Association between health-related hope and adherence to prescribed treatment in CKD patients: multicenter cross-sectional study
Source: BMC Nephrol. 2020 Oct 31;21:453. doi: 10.1186/s12882-020-02120-0 (PMC7603681; doi:10.1186/s12882-020-02120-0)
Supplement: Supplementary file 5 — Additional file 5: Table S4. The associations between HR-Hope and serum phosphorus levels. [file 12882_2020_2120_MOESM5_ESM.docx]

# **Table S4. Associations between HR-Hope and serum phosphorus levels^a^**

|  | Serum phosphorus (n = 422), Mean difference, mg/dL (95%CI) | | | | | | | | | | | | | | |
| --- | --- | --- | --- | --- | --- | --- | --- | --- | --- | --- | --- | --- | --- | --- | --- |
|  | Unadjusted | | | | | Adjusted 1^b^ | | | | | Adjusted 2^c^ | | | | |
| **HR-Hope** |  | | | | |  | | | | |  | | | | |
| *per 10 points* | -0.07 | | | | | -0.01 | | | | | 0.00 | | | | |
|  |  | (-0.15 | - | 0.01) |  |  | (-0.08 | - | 0.06) |  |  | (-0.07 | - | 0.07) |  |
| *per 1 SD* | -0.13 | | | | | -0.02 | | | | | 0.00 | | | | |
|  |  | (-0.27 | - | 0.02) |  |  | (-0.14 | - | 0.10) |  |  | (-0.13 | - | 0.12) |  |

^a^General linear models were used to estimate mean differences in serum phosphorus values.

^b^Adjusted for age, gender, stage of renal disease, performance status, presence of family, work status, and categories of number of prescribed phosphate binders

^c^Adjusted for the covariates listed in footnote b, and also for primary renal disease, diabetes, coronary artery disease, and cerebrovascular disease

HR-Hope: health-related hope; 95%CI: 95% Confidence interval
