# Supplementary material for: Association between health-related hope and adherence to prescribed treatment in CKD patients: multicenter cross-sectional study
Source: BMC Nephrol. 2020 Oct 31;21:453. doi: 10.1186/s12882-020-02120-0 (PMC7603681; doi:10.1186/s12882-020-02120-0)
Supplement: Supplementary file 6 — Additional file 6: Table S5. The associations between HR-Hope and serum potassium levels. [file 12882_2020_2120_MOESM6_ESM.docx]

# **Table S5. Associations between HR-Hope and serum potassium levels^a^**

|  | Serum potassium (n = 453), Mean difference, mEq/L (95%CI) | | | | | | | | | | | | | | |
| --- | --- | --- | --- | --- | --- | --- | --- | --- | --- | --- | --- | --- | --- | --- | --- |
|  | Unadjusted | | | | | Adjusted 1^b^ | | | | | Adjusted 2^c^ | | | | |
| **HR-Hope** |  | | | | |  | | | | |  | | | | |
| *per 10 points* | 0.01 | | | | | 0.02 | | | | | 0.02 | | | | |
|  |  | (-0.03 | - | 0.04) |  |  | (-0.02 | - | 0.05) |  |  | (-0.02 | - | 0.05) |  |
| *per 1 SD* | 0.02 | | | | | 0.04 | | | | | 0.04 | | | | |
|  |  | (-0.05 | - | 0.08) |  |  | (-0.03 | - | 0.10) |  |  | (-0.03 | - | 0.10) |  |

^a^General linear models were used to estimate mean differences in serum potassium values.

^b^Adjusted for age, gender, stage of renal disease, performance status, presence of family, work status, and potassium binders

^c^Adjusted for the covariates listed in footnote b, and also for primary renal disease, diabetes, coronary artery disease, and cerebrovascular disease

HR-Hope: health-related hope; 95%CI: 95% Confidence interval
